# Group brief cognitive behavioral therapy for suicide prevention compared to dialectal behavior therapy skills group for military service members: a study protocol of a randomized controlled trial

**DOI:** 10.1186/s12888-023-05282-x

**Published:** 2023-12-05

**Authors:** Justin C. Baker, Shawna Grover, Laura H. Gunn, Cindy Charles, Heather Rikli, Michael J. Franks, Lauren R. Khazem, Sean Williams, Ennio Ammendola, Cherita Washington, Marquita Bennette, Austin Starkey, Kelly Schnecke, Shannon Cain, Craig J. Bryan, Robert J. Cramer

**Affiliations:** 1 The Ohio State University Wexner Medical Center, 1960 Kenny Road, Columbus, OH 43210 USA; 2 Naval Medical Center Portsmouth, 620 John Paul Jones Cir, Portsmouth, VA 23708 USA; 3 University of North Carolina at Charlotte, 9201 University City Blvd, Charlotte, NC 28223 USA; 4 Louisiana State University, 236 Audubon Hall, Baton Rouge, LA 70803 USA

**Keywords:** Military, Group therapy, Suicide prevention, Emotion regulation, Coping self-efficacy, Cognitive-behavioral therapy, Dialectical behavior therapy

## Abstract

**Background:**

Suicide is a pressing matter for the military. Not only does it pose a health risk, but suicide also compromises operational readiness. Despite provision of suicide prevention clinical best practices, the Department of Defense suffers several challenges (e.g., clinician shortages) limiting the agency’s ability to effectively respond to service member suicide. Implementation of evidence-based suicide-specific group therapy is a possible solution to service member well-being needs and system challenges. Service members can also gain coping skills useful beyond managing suicidal thoughts and behaviors.

**Methods:**

This 2-arm non-inferiority randomized controlled trial compares a group therapy format of Brief Cognitive Behavioral Therapy (i.e., G-BCBT) with Dialectical Behavior Therapy (DBT) Skills Group. Both therapies are delivered in-person at a United States Naval Medical Center. Participants (N = 136) are active-duty service members with recent suicidal thoughts or suicidal behavior. Evaluation features electronically delivered questionnaires at baseline, after each treatment session, and at 3- and 6-month follow-up.

**Discussion:**

The primary outcome concerns G-BCBT impacts on suicidal ideation. Secondary outcomes of interest are suicide attempt, psychological distress (e.g., symptoms of depression, anxiety), and self-regulatory skills (e.g., emotion regulation). We also examine self-regulatory skills as treatment moderators. Clinical trial strengths and limitations are reviewed.

**Trial registration:**

This study was registered at Clinicaltrials.gov (protocol NCT05401838).

**Supplementary Information:**

The online version contains supplementary material available at 10.1186/s12888-023-05282-x.

## Background

Reducing the proportion of military service members who die by suicide or make a suicide attempt remains a public health and national security crisis in the United States (US) [1]. The suicide rate for US active-duty service members is significantly greater (24.3/100,000 persons) [2] than that for US civilians (14.1/100,000) [3]. Suicide deaths are disproportionately represented among young (< 30) male service members across all branches and components [2] and are ranked among the top three causes of death for all active-duty military personnel [4]. Meta-analyses assessing the global prevalence of suicidal behaviors among military personnel worldwide demonstrated a pooled prevalence of 12% and 14% for suicidal ideation and attempt, respectively [5]. Contextually adapted treatments targeting suicidal thoughts and behaviors (STBs) for active-duty military personnel are needed to reduce elevated rates of suicide among service members [6, 7].

Meta-analytic reviews have identified several suicide prevention interventions developed specifically for military personnel that have strong supporting evidence for reducing STBs in service member populations [8]. These treatments include Brief Cognitive Behavioral Therapy (BCBT) [9] for suicide prevention and the Collaborative Assessment and Management of Suicidality (CAMS) [10]. The Department of Veterans Affairs and Department of Defense (Department of VA/DoD) [11] created suicide clinical practice guidelines aligning with best therapeutic practices. For instance, Cognitive-Behavioral Therapy (CBT) and Dialectical Behavior Therapy (DBT) were identified as individual therapies with best supporting evidence to reduce STBs. Availability of mental health providers trained in these evidence-based suicide prevention interventions is limited, however, throughout the Defense Health Agency (DHA) [12]. Many providers also continue to use contraindicated interventions (i.e., contracting for safety) at high rates [13]. The recent Suicide Prevention and Response Independent Review Committee [14] identified a number of barriers to effective suicide prevention care for active duty service members. Among these were provider shortages, onboarding/hiring delays, and the need to enhance evaluation of suicide prevention initiatives.

This paper outlines a clinical trial testing a possible solution to overcome these barriers and increase service member access to care through a group adaption of BCBT. Group therapy treatment formats offer a way to implement evidence-based mental health interventions to increase both the number of service members in care and the quality of care they receive [15, 16]. Group therapy can also build cohesion among patients, leading to better outcomes [17]. Below we outline a randomized controlled trial (RCT) protocol for a new Group Brief Cognitive Behavioral Therapy (G-BCBT). G-BCBT is adapted from individual therapy BCBT [7, 9], and compared to Dialectical Behavior Therapy (DBT) skills group [18] in the present protocol.

### Group therapy interventions for military suicide

A review of available literature highlights that previous studies investigating the efficacy of group treatment for suicide are scarce, especially among active-duty military personnel [19]. Previous group therapy studies most notably incorporate DBT elements [20, 21], elements from CAMS [10], as well as psychoeducation and problem-solving elements [22, 23]. Group CBT for other conditions such as post-traumatic stress [24] have also been examined for impacts on suicide. Veterans receiving services from their local VA facilities appear to be the most studied, with a few studies demonstrating the possible effectiveness of group therapy for veteran suicide in both inpatient and outpatient settings [20, 25, 26]. In the following sections, we detail this literature to establish the groundwork for G-BCBT.

#### Dialectical behavior therapy

Many of the group designs offer some of the tenets of DBT distress tolerance skills but did not adhere to the time commitment of full model DBT [20, 21]. For example, Goodman et al.’s [25] Project Life Force trial examined feasibility, acceptability, and exploratory outcomes. Researchers found a significant decrease in intensity of suicidal ideation, as well as in individuals finding “deterrents” to suicide. Furthermore, Denckla et al. [20] investigated the efficacy of a drop-in outpatient distress tolerance skills group for veterans, finding a reduction in crisis events based on group participation. Finally, Anestis et al. [21] utilized a traditional DBT application with teenagers in a military style boot camp with a control group of those receiving treatment as usual. After treatment, adolescents randomized to receive DBT displayed greater improvements in emotional regulation, but not distress tolerance, compared to the control group. The study included suicidality within the emotional regulation category in posttreatment measures.

#### Cognitive-behavioral therapy

CBT frameworks comprised the bulk of the other studies used with military populations [7, 23, 24]. Most relevant to the present trial, Rudd et al. [7] utilized the individual format of BCBT for outpatient active-duty soldiers with a primary focus on suicide attempts. Soldiers who received BCBT were 60% less likely to make a subsequent suicide attempt for up to two years, with significant between-treatment effects emerging as early as three months. Service members receiving BCBT were also less likely to be medically separated from the military during follow-up. Regarding group therapy, Bryan et al. [24] studied Cognitive Processing Therapy against Present Centered Therapy in group intervention formats among active-duty soldiers who were diagnosed with post-traumatic stress disorder (PTSD). Service members in both groups experienced a decrease in suicidal ideation.

#### Collaborative assessment and management of suicidality

Other types of group interventions beyond DBT and CBT center on the CAMS treatment approach [10]. For instance, Johnson et al. [27] utilized CAMS elements for military veterans. Authors found that, while no structured group protocol was utilized, post-hospitalization CAMS-based group therapy is an acceptable intervention for treating suicidal ideation among veterans in a real-world clinical setting. A pilot study examining the effectiveness of CAMS-group compared to treatment as usual demonstrated good satisfaction with the treatment, a sense of cohesion with other group members, and reduced psychological symptoms [28]. Veterans in either treatment reported similar modest reductions in STBs. Finally, O’Connor et al. [29] completed a 3-year follow-up study to determine the long-term effects of a suicide prevention-focused group therapy. Importantly, higher group cohesion was associated with a reduced likelihood of inpatient psychiatric hospitalizations and greater engagement in outpatient mental health services.

#### Other unstructured group therapies

Beyond CAMS, DBT and CBT, Simons et al. [26] investigated a weekly drop-in support group comprising open topics (i.e., no agenda or set protocol) supplemented with psychoeducation. Veterans participating in the intervention group saw an 81% reduction in suicidal ideation. Finally, Gebhardt et al. [30] developed a single-session psychoeducation group addressing suicide risk for veterans on a psychiatric unit. The study found that veterans’ acceptability of the group was high, and veterans were more hopeful and motivated to learn new skills to cope with suicidal thoughts and behaviors in a group setting.

### BCBT treatment moderators

**Fig. 1:**
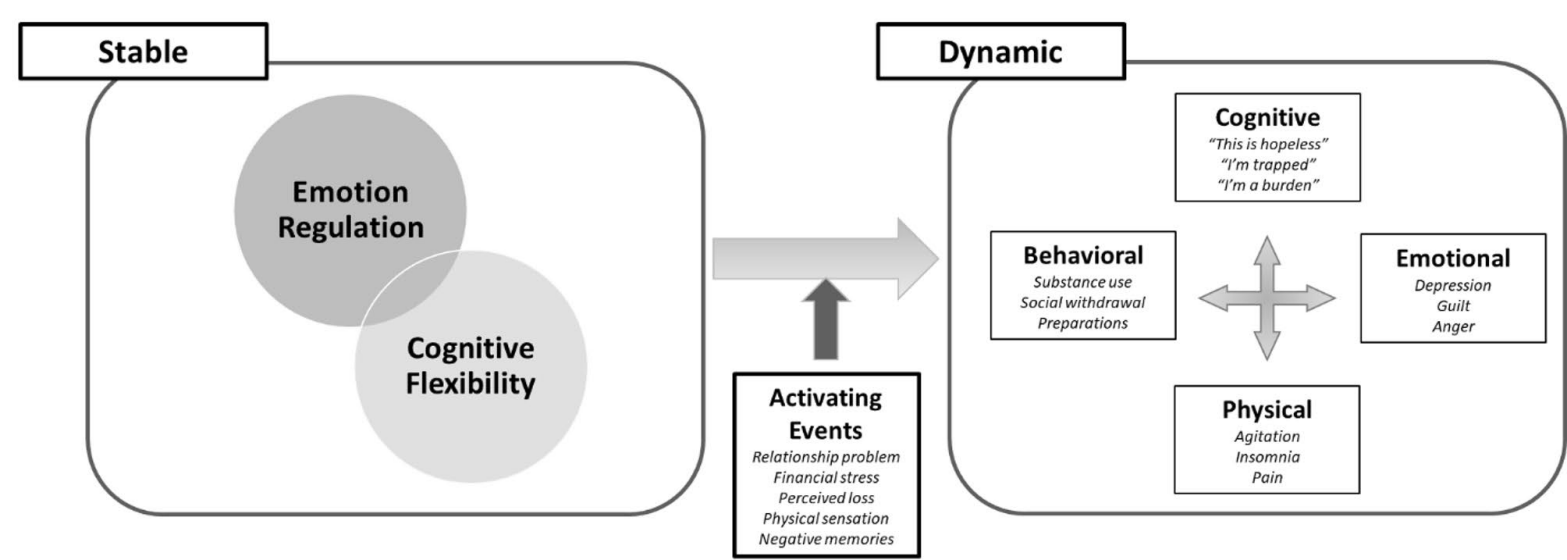
The suicide mode

Conceptual and empirical underpinnings of BCBT provide rationale for selection of possible treatment moderators. BCBT adheres to the fluid vulnerability theory, which describes the ephemerality of heightened suicide risk [31]. The fluid vulnerability theory is a diathesis-stress model, consistent with ideation-to-action theories of suicide [32], that describes suicide risk as ever fluctuating, time-limited, and impacted by existing vulnerabilities and acute risk factors of the individual. Briefly, this person-environment model holds that a person’s suicidal state is a result of deficits in emotion regulation and cognitive flexibility interacting with an activating event (i.e., relationship breakup, financial strain, legal difficulties, etc.). Once activated, an individual may respond with problematic behavioral, cognitive, emotional, and physical processes defining one’s unique suicide mode (see Fig. 1). Importantly, deficits in self-regulatory abilities, leading to emotion dysregulation, are linked with suicide ideation and transition of ideation to attempts [33]. Moreover, BCBT impacts on reduced suicidal behavior has been theorized to be a function of training in emotion regulation, cognitive reappraisal, and problem-solving skills [34].

The suicide mode and accompanying empirical data, therefore, raise the possibility that one’s self-regulatory abilities (i.e., emotion regulation, coping related-beliefs, and behavioral control) or lack thereof may moderate BCBT treatment outcomes. A number of empirical findings support this promise. For instance, in one study, the most commonly identified reason for service members’ suicide attempts was to seek relief from emotional distress [35]. A subsequent study showed that service members who experienced reductions in emotional distress after their first suicide attempt were much more likely to attempt suicide again as they learned attempting suicide to be an effective, albeit maladaptive, coping strategy to alleviate their unwanted emotional distress [36]. Importantly, in that same study, individuals who received treatment following their first suicide attempt were less likely to make a subsequent suicide attempt. Notably, a recent review of military suicide concluded that emotion dysregulation is a robust suicide risk factor uniquely impacting service members [37]. Regarding coping-related beliefs, coping self-efficacy (CSE) is particularly relevant to treatment-seeking active-duty service members at the site of this RCT [38]. All three CSE domains (i.e., problem-focused, thought stopping, and social support beliefs) were significantly and negatively associated with 12-month suicide ideation, future perceived likelihood of a suicide attempt, and a total metric of lifetime suicide-related behavior. After controlling for various military (e.g., number of deployments) and mental health (e.g., depressive symptoms) factors, thought stopping beliefs accounted for unique variance in all suicide outcomes. Finally, behavioral inhibition has been linked to suicide ideation through unhealthy emotion regulation skills in a non-military sample [39].

### Group risk reduction intervention therapy project aims and hypotheses

We named this G-BCBT focused clinical trial project Group Risk Reduction Intervention Therapy (Project GRRIT). The overall goal of Project GRRIT is to test the effectiveness of G-BCBT in a sample of treatment-seeking active-duty service members when compared to DBT.

Aim 1: To implement a group format of BCBT for its impact on suicidal behavior among active-duty military service members.

#### Hypothesis 1a

Service members randomized to the G-BCBT condition will see non-inferior reductions in suicidal ideation at 6-month follow-up (primary outcome point) compared to service members randomized to the DBT skills training condition. We will also evaluate 3-month follow-up.

#### Hypothesis 1b

Service members randomized to the G-BCBT condition will be no more likely to make a suicide attempt during the 3- and 6- month follow-up periods, compared to service members randomized to the DBT skills training condition.

Aim 2: To assess the relationship between G-BCBT and self-regulatory factors.

#### Hypothesis 2a

Service members randomized to the G-BCBT condition will show non- inferior increases in self-regulatory characteristics (i.e., coping self-efficacy, behavioral inhibition, and emotion regulation skills) at 3- and 6-month follow-up, compared to service members randomized to the DBT skills training condition.

#### Hypothesis 2b

We will explore self-regulatory characteristics (i.e., coping self-efficacy, behavioral inhibition, and emotion regulation skills) as moderators of intervention effects on suicidal ideation.

## Methods

### Design

We propose a 4-year, 2-arm phase III randomized controlled trial of G-BCBT compared to a DBT skills group. G-BCBT is a new adaptation of the existing BCBT protocol [9] administered in a group format (see description below). DBT’s efficacy in a group format is already well supported [20, 40, 41]; therefore, we anticipate G-BCBT to be at least as equally effective accounting for the 2-arm non-inferiority study design. The trial will follow the Standard Protocol Items: Recommendations for Interventional Trials (SPIRIT) protocol and is registered at clinicaltrials.gov (NCT05401838). The study protocol was approved by the Navy Medical Center Portsmouth (NMCP) Institutional Review Board (IRB) in compliance with all applicable Federal regulations governing the protection of human subjects. Current protocol version: Version 1.5 July 20th, 2023.

### Study setting

Naval Medical Center Portsmouth (NMCP) is the selected study site for this randomized controlled trial. NCMP stand as the Navy’s first and longest-standing hospital in continuous operation [42]. It currently provides care to approximately 420,000 active-duty members, family members, and retirees in the Hampton Roads geographic region of Virginia [42]. A total of 136 treatment seeking active-duty service members will be enrolled in the study at NMCP in Portsmouth, Virginia. The patient population is primarily Navy sailors, although all branches of the military are represented in the NMCP patient population and will be eligible to participate.

### Eligibility criteria

Study participants meet inclusion criteria if they are (1) active duty service members, (2) between the ages of 18 to 65, (3) of treatment-seeking status in outpatient mental health or substance abuse rehabilitation clinics, and/or inpatient psychiatry discharge, or other NMCP primary care and surrounding outpatient clinics (4) report current (within the past week) suicide ideation (e.g., score greater than 2 on the Scale for Suicide Ideation) and/or a suicide attempt within the past month (e.g., as assessed by the Self-injurious Thoughts and Behaviors Interview-Revised [SITBI-R] [43]), (5) able to understand and speak English, and (6) able to complete the informed consent process. Persons will be excluded from study enrollment if they have a psychiatric or medical condition that precludes the ability to provide informed consent or participation in outpatient treatment (e.g., psychosis, mania, acute intoxication). Retired service members and family/dependents will also be excluded.

### Interventions

Participants will be randomized to receive either G-BCBT or DBT, delivered in group format. DBT skills group [44] was selected as the comparator treatment as it is considered a gold standard treatment for problems such as self-injury and suicidal behavior [45]. DBT skills group has demonstrated significant reductions in frequency of suicidal events and improved emotion regulation skills compared to treatment as usual for those diagnosed with borderline personality disorder [46]. As a result, DBT is considered only one of three recommended psychotherapeutic interventions for the reduction of suicidal behavior for military service members and veterans [11]. Participants will be allowed to be engaged in other mental health treatments during the course of the study (e.g., individual therapy, medication management).

#### Group-brief cognitive behavioral therapy (G-BCBT)

Adhering to a cognitive behavior therapy paradigm, G-BCBT targets the following hypothesized mechanisms of action—emotion dysregulation and cognitive rigidity—that are conceptualized as underlying suicide specific vulnerabilities. Participants assigned to G-BCBT will undergo an initial individual intake session followed by 12 group therapy sessions scheduled for 90 min on a weekly basis. Sessions are organized into three phases: (1) emotion regulation, (2) cognitive flexibility, and (3) relapse prevention. The intake session consists of a tailored individual session for each participant, which includes the narrative assessment, development of the person’s suicide mode, and crisis response plan. The narrative assessment is a patient-centered suicide risk assessment, whereby the therapist instructs the patient to retell what transpired during their recent suicide crisis. Next, the therapist and patient collaboratively develop a person’s suicide mode. The suicide mode captures a person’s predisposed vulnerabilities and risk factors, activating stressor, and skills deficits in the following four domains: cognitions, emotions, behaviors, and physical reactions. The development of the suicide mode will function as an individualized conceptualization for the participant linking the specific skills, they will learn through group therapy, to address identified difficulties as it pertains to their specific skills deficits. Towards the end of the session, the therapist will introduce the crisis response plan and develop with the participant an individualized plan that identifies targeted skills the participant can use in a crisis. The first session may also include means safety counseling to help reduce access to potentially lethal means of suicide for the participant. The provider will also use this session to assess the individual’s appropriateness for group therapy.

Phase I of the group therapy sessions will focus on improving emotion regulation and consist of sessions 1–5. Emotion regulation skills will introduce coping strategies like mindfulness and relaxation training to help increase participants’ ability to deescalate during a crisis. Phase II, which includes sessions 6–10, will target improving cognitive flexibility. This will be accomplished using cognitive restructuring tasks that will focus on identifying core suicidogenic beliefs (e.g., hopelessness, burdensomeness, unlovability) and teach group participants to reappraise their maladaptive thoughts into more helpful thoughts. Phase III of treatment (session 11–12) focuses on relapse prevention. The relapse prevention task consists of demonstrating mastery of skills learned in group therapy as participants imagine themselves using their new skills to avoid a suicidal episode. Past and potential future suicide events are explored.

#### Dialectical behavior therapy (DBT)

Participants randomized to receive DBT will receive an individual intake session followed by 24 weekly group therapy sessions each lasting 90 min. DBT Skills group follows an evidence-based manualized protocol [18]. Four core skill domains are addressed in DBT: (1) mindfulness (i.e., learning to stay in the present moment); (2) distress tolerance (i.e., learning generalized stress reduction strategies to decrease the intensity and frequency of high acuity situations); (3) emotion regulation (i.e., strategies to regulate baseline experience and expression of affect); and (4) interpersonal effectiveness (i.e., establishing healthy relationships). Three treatment phases occur. Phase I (8 weeks) entails two weeks of mindfulness orientation followed by six weeks of distress tolerance modules. Phase II (9 weeks) entails a two-week mindfulness module with seven weeks of emotion regulation skill building afterward. Phase IIII (7 weeks) includes a two-week mindfulness and orientation module, followed by five weeks of interpersonal effectiveness training. Mindfulness is a foundational element preceding other skill building domains.

Table 1 contains a comparison of intervention treatment elements and characteristics. DBT and G-BCBT are differentiated in several ways. DBT’s foundation is routed in mindfulness and emotion regulation as reflected in the mindfulness skill domain being repeated throughout the group. Emotion regulation, including mindfulness, is one coping skill domain covered by G-BCBT, which adds tailored crisis response planning and cognitive flexibility/reappraisal grounded in CBT theory [9]. DBT skills group training and G-BCBT also vary in session length, and therefore the resources required to provide the services (e.g., provider time). G-BCBT requires half the length of sessions, reducing the required resources and increasing the number of service members able to access evidence-based suicide-specific care.

**Table 1:** DBT Group Skills Training versus G-BCBT

Treatment Element or Characteristic	G-BCBT	DBT Group
Suicide risk screening	X	X
Narrative assessment	X	
Crisis response plan/safety plan	X	X
Means safety counseling	X	
Weekly monitoring of suicide risk	X	
Psychiatric symptom management	X	
Psychoeducation: suicide as a deficit in self-regulation	X	X
Emotion regulation & mindfulness skills training	X	X
Cognitive flexibility skills training	X	
Relapse prevention task	X	
Interpersonal effectiveness skills training	X	
Distress tolerance skills training	X	
Length of time of each session	90 min	90 min
Number of sessions	12	24

Notes: G-BCBT = Group Brief Cognitive Behavioral Therapy; DBT = Dialectical Behavior Therapy Skills Group

### Training, supervision, and monitoring of study clinicians

Two study clinicians were hired to deliver both group treatments. Study clinicians were selected based on the following criteria: (1) eligible to receive clinical privileges at NMCP, (2) previous experience working with military populations, (3) tolerance for and ability to work with patients who have significant emotional distress in an accepting, nonjudgmental manner, (4) ability to handle crises and crisis patients and is able to hospitalize patients if necessary, (5) prior experience conducting group therapy, (6) willingness to receive training and supervision and a desire to learn new approaches to brief risk management, and (7) conscientiousness in documentation of case files.

Both study clinicians completed a two-day BCBT workshop and a one-day Crisis Response Planning workshop taught by investigators of this study. Study clinicians also completed a four-day workshop in DBT skills introduction from Behavioral [47]. Trainings consisted of didactic instruction, live supervised role plays with feedback, video demonstration, and assigned reading materials. Study clinicians then completed mock sessions in both treatment modalities and received fidelity ratings and live feedback during weekly supervision sessions. Study clinicians also received training in site-specific policies and procedures, including use of the electronic medical record, documentation requirements, and administrative procedures unique to the military context and U.S. Navy.

Study clinicians completed all onsite requirements for full licensure and credentialing to be independent licensed providers within the NMCP outpatient mental health clinic. Clinicians will participate in weekly supervision and fidelity review with clinical lead investigators of this study. All individual and group therapy sessions for both conditions are recorded for purposes of supervision and fidelity monitoring by the investigators. All initial therapy sessions will be reviewed until clinicians reach a score of 85% or higher on G-BCBT fidelity checklists for two consecutive courses of group therapy, following similar procedures in previous BCBT RCTs [7, 48]. G-BCBT fidelity checklists are modified for group delivery from currently available individual BCBT fidelity checklists [9]. DBT fidelity will be monitored using the DBT-California Competency Scales (DBTCCs) Scoring Principles [49]. DBTCCSs items are rated 0 to 6 (0 = poor, 6 = excellent). An average score of 4 across all items for a total score of 48 is considered as passing. Once fidelity has been established, a random selection of no less than 20% of sessions will be reviewed for fidelity to ensure fidelity maintains above 85% accuracy for G-BCBT and above a total score of 48 for DBT. If a clinician falls below approved fidelity ratings, additional training and monitoring of sessions will occur to ensure the clinician achieves sufficient fidelity. Fidelity reviews will be completed by trained independent evaluators. Independent evaluators will review recorded sessions for fidelity according to the benchmarks outlined above and provide timely feedback to the study clinicians.

### Outcomes

The primary study outcome is suicidal ideation as measured by the Scale for Suicide Ideation [50]. We selected this primary outcome for three reasons. First, suicidal ideation is a central mechanism within the BCBT suicide mode [9], thereby serving as a proximal indicator of treatment effectiveness. Second, compared to treatment as usual (TAU), individual BCBT has demonstrated larger reductions in worst-point and current suicidal ideation at 6-month follow-up [7]. We plan to assess replication of this finding for the G-BCBT format. Third, from an ideation-to-action perspective [51], suicide ideation is a critical element in the pathway from many suicide risk factors to attempt or death. Therefore, we treated suicide ideation as a proximal outcome to informed intervention efforts toward the goal of breaking the chain leading to suicide attempt or death. Secondary outcomes include suicide attempt and psychological distress. BCBT is associated with reduction in suicide attempt frequency among service members [7], so we therefore seek to assess this finding within the group format as well. A secondary goal of our group therapy intervention is to improve mental health; therefore, we selected a range of common domains of psychological distress that are either strongly linked with suicide and/or very common for service members. These include symptoms of anxiety, depression, insomnia, hopelessness, and post-traumatic stress. Suicidal cognitions, or a suicidal belief system, is also an indicated precursor or risk factor for STBs among military service members [52]. As such, we will assess suicidal cognitions along with psychological distress secondary outcomes. Self-regulatory characteristic treatment moderator selection was driven by BCBT and cognitive-behavioral theories and research (see background section for details).

### Sample size

For the proposed non-inferiority hypotheses, intent-to-treat analysis will be used to handle effects of missingness. Assuming 80% power, 5% significance level, and 25% attrition informed by a prior BCBT trial [7], as well as equal group variances and equal group allocation, then a minimum sample size of 136 service members (n = 68 per group) is needed. The study is powered to detect a non-inferiority minimal clinical difference in means between groups of 4.5 points on the SSI. This metric is based on prior military suicide intervention clinical trials suggesting an anticipated difference in means between groups of 1.5 points [7, 34] on the SSI over the study period for two equal-sized independent samples (i.e., G-BCBT and DBT) with an equal standard deviation of 6 [7, 34] for participants in each group.

### Recruitment

The study population for the RCT will be recruited and referred from the following NMCP treatment clinics: (1) Outpatient Mental Health Clinic, (2) Outpatient Mental Health ER Direct Access, (3) Inpatient Psychiatric Discharge Referral, (4) NMCP Primary Care Clinic, and (5) Outpatient Branch Health Clinics. Prospective participants will be referred to our research staff if deemed appropriate based off inclusion/exclusion criteria by referring mental health providers as part of routine care and/or as a part of their discharge plan from inpatient psychiatric treatment. Research staff will attend weekly clinic team meetings, weekly hospital discharge planning meetings, and daily morning hospitalization reports to identify potential participants.

The study research coordinator will meet with each possible participant in a private office within a treatment clinic at NMCP. The coordinator will review and explain study information, including, but not limited to, anonymity and confidentiality, study clinical and data collection procedures (e.g., random assignment), possible risks and benefits, and data usage. Service members will be provided the opportunity to review the consent documentation and ask questions. Service members will also be informed that they can discuss the study information with any family members present at the time of recruitment. Those willing to participate will sign consent documentation. The research clinicians for this RCT are individually licensed master’s level therapists. Clinicians completed all credentialing requirements to practice at NMCP as hired contractors employed through the University of North Carolina at Charlotte.

### Randomization/allocation

**Fig. 2:**
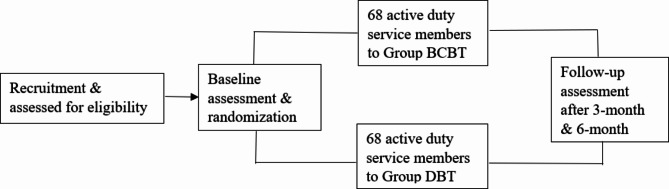
Planned participant experience

Those who complete the informed consent process will be assigned a unique, sequential identifier (ID; e.g., 1001, 1002, etc.) prior to eligibility screening. Those who are eligible to participate in the study will proceed to baseline data collection. Following baseline survey completion, the participant’s sex and suicide attempt history will be used to stratify across one of four sex by history of suicide attempt strata (i.e., male without suicide attempt history, female without suicide attempt history, male with suicide attempt history, and female with suicide attempt history). Note that participant IDs corresponding to eligibility screen failures will not be used in the randomization process nor counted towards the 136-participant sample size calculated for this study. Upon identification of the stratum, participants will be randomly assigned to either treatment condition (G-BCBT, DBT) based on stratum-specific sequences/schedules pre-defined by team’s biostatistician and co-investigator ahead of trial commencement using the *blockrand* package in R v1.5 [53]. Although strata are not anticipated to be equal-sized, each of the stratum-specific schedules are randomized to achieve balance between each treatment condition. See Fig. 2 for the diagram outlining participant recruitment, screening, treatment randomization, and follow-up schedules.

### Blinding

To minimize bias, Co-I Gunn (biostatistician) will be blinded to intervention condition during data collection and preparation for analyses; the dataset will be cleaned by other project staff and intervention condition will be referred to only as intervention A or B. Participants will also be blinded to which is the intervention condition and which is the comparison condition. They will know that one treatment condition is twice as long as the other.

### Data collection methods

#### Schedule of assessments

The proposed assessment schedule for all key study variables is included in Table 2. The assessment battery can be obtained from the corresponding author by email contact. Primary and secondary outcomes will be assessed at baseline, during treatment, and at 3- and 6-month follow-ups. All assessments will be completed by the participant via self-report using Qualtrics, an online survey database. The baseline assessment, which will include demographic variables (age, sex, gender, race, ethnicity, marital/relationship status, branch of service, branch component, highest rank achieved, deployment history, prior suicide attempts, current diagnoses, and medication usage) and other self-report assessments (see Table 2) will be completed following informed consent and the eligibility screening in person with the research coordinator. During treatment measures will be completed via the participants smartphone, tablet, iPad, or computer at home or in the waiting room prior to each therapy session. All follow-up assessments will be completed during off-duty hours at the service members’ convenience.

**Table 2:** Assessment Schedule

Measures	BL	Tx	3 Mo	6 Mo
Demographics	X			
**Primary Outcome**:				
Scale for Suicidal Ideation	X	X	X	X
**Secondary Outcomes**:				
Self-Injurious Thoughts and Behaviors Interview- Revised	X	X	X	X
Behavioral Health Data Portal and Medical Record Review	X		X	X
Patient Health Questionnaire-9	X	X	X	X
Generalized Anxiety Questionnaire-7	X	X	X	X
Insomnia Severity Index	X	X	X	X
Beck Hopelessness Scale	X	X	X	X
PTSD Checklist-5	X	X	X	X
Suicide Cognitions Scale-Revised	X	X	X	X
**Treatment Moderators**:				
Coping Self-Efficacy Scale	X		X	X
Monetary Choice Questionnaire	X		X	X
Emotion Regulation Questionnaire	X		X	X
**Treatment Process Variables**:				
Working Alliance Inventory-Short Form		X		
Credibility/Expectancy Questionnaire	X			
**Covariates and Descriptors**:				
Traumatic Brain Injury-4	X		X	
Brief Reasons for Living Inventory	X		X	

Notes: BL = Baseline; Tx = During and post-treatment; 3 Mo = 3-month follow-up; 6 Mo = 6-month follow-up

#### Participant retention

We will utilize a number of strategies shown to enhance retention in past and current intervention trials. First, we plan a flexible communication strategy that includes recording up-to-date contact information, tracking deployments or relocations, and obtaining consent to schedule follow-up assessments and provide attendance reminders via text messaging or email. To enhance our ability to contact participants, we will obtain at least two verifiable contact persons for the patient at baseline, at least one of whom is a parent, family member, or a significant other. These individuals should have the ability to know the location of the participant despite potential station changes or deployments. Second, in line with empirical research showing that use of gift card incentives enhances participant retention in health survey research [54], we will provide a modest incentive for all participants, namely a $50 Amazon e-gift card at both 3- and 6-month follow-up. Third, in instances of lack of communication from follow-up participants, we will contact participants to provide the opportunity to complete self-report questionnaires with the assistance of a study team member via a telephone or secure online platform or conduct (e.g., HIPAA-compliant videoconferencing platform). We will specifically focus our efforts at continual communication with any “lost to follow-up” patients. Finally, to enhance likelihood of participant follow-up, we will use a follow-up scheduling strategy of increased frequency of assessments and a shorter time commitment for assessment. That is, the short (30–45 min) assessments at 3- and 6-month follow-up should ensure continued participant engagement because it will remain fresh in their minds.

#### Primary outcome: suicidal ideation

Suicide ideation will be measured with the Scale for Suicide Ideation (SSI) [50]. The SSI contains 19 self-report items assessing thoughts and attitudes about suicide, as well as steps taken to prepare for a suicide attempt, that have occurred within the past week. The SSI is included as a recommended measure in the NIMH PhenX Toolkit [55]. The SSI demonstrates strong reliability and associations with suicide measures when used with military personnel [56].

#### Secondary outcomes: suicide attempts

Suicide attempts will be defined as intentional self-injurious behavior for which there is evidence of intent to die as a result of the behavior. Physical injury or tissue damage is not required to be classified as an attempt [57]. We will measure suicide attempts using the SITBI-R, a structured clinician administered interview that assesses the timing, method and other features of suicide attempts and other self-injurious behaviors [43]. The SITBI-R has demonstrated good construct validity. As has been done in prior research, the SITBI-R will be adapted to a self-report format for this study. Review of the participant’s medical records, to include the US Military’s Behavioral Health Data Portal, will additionally be conducted to identify suicide attempts that might have been missed during SITBI-R administrations (e.g., participants who drop out early or miss follow-up assessments).

#### Secondary outcomes: depression

Depression will be quantified with the Patient Health Questionnaire-9 (PHQ-9) [58]. The PHQ-9 contains nine self-report items measuring depression symptom severity. It has demonstrated high internal consistency among treatment-seeking service members, and construct validity in a clinical sample [38].

#### Secondary outcomes: anxiety

Anxiety will be assessed with the Generalized Anxiety Disorder-7 (GAD-7) [59], which contains 7 self-report items capturing anxiety symptoms. The GAD-7 has demonstrated high internal consistency among treatment-seeking service members [38], and construct validity in a clinical sample [59].

#### Secondary outcomes: sleep difficulties

Sleep difficulties will be measured with the Insomnia Severity Index (ISI) [60]. The ISI has 7 self-report items capturing subjective sleep quality. The ISI demonstrates high internal consistency and strong validity among treatment-seeking active-duty service members [61].

#### Secondary outcomes: hopelessness

Hopelessness will be assessed with the Beck Hopelessness Scale (BHS) [62], a 20-item true/false self-report survey that measures the intensity of negative expectations about the future. The BHS possesses high internal consistency and appropriate construct validity in military samples [63].

#### Secondary outcomes: posttraumatic stress symptoms

Posttraumatic stress disorder (PTSD) symptoms will be measured with the Primary Care PTSD Screen for DSM-5 (PC-PTSD-5) [64, 65]. The PC-PTSD-5 begins with an item that assess lifetime exposure to traumatic events. If participants deny having any trauma exposure, no further questions are asked and the survey is scored a 0. If participants endorse exposure to a traumatic event, 5 additional yes/no questions are presented to the participant asking how the traumatic event has impacted them over the past month. PC-PTSD-5 items correspond with primary PTSD symptom clusters according to the DSM-5. PC-PTSD-5 has demonstrated high sensitivity, specificity, and efficiency in US Veteran samples [64, 65].

#### Secondary outcomes: suicidal beliefs

Suicidal beliefs will be assessed via the Suicide Cognitions Scale-Revised (SCS-R) [52], a 16-item self-report survey providing a total score of suicide-related thinking patterns (e.g., “No one can help solve my problems”. The SCS-R total score possess high internal consistency and associations with depression and suicide outcomes in a clinical sample service members and veterans.

### Treatment moderators

#### Coping self-efficacy

Coping skill related-beliefs will be assessed with the Coping Self-Efficacy Scale (CSES) [66], a 13-item self-report survey yielding three domains: problem-focused coping, using social support, and stopping negative thoughts/emotions. All subscales demonstrate high internal consistency and positive correlations with suicide outcomes in a sample of active-duty service members [38].

#### Behavioral inhibition

Behavioral inhibition will be assessed with the Monetary Choice Questionnaire (MCQ) [67, 68]. The MCQ is a 21-item self-administered questionnaire in which the respondent chooses between a smaller, immediate monetary award and a larger, delayed monetary reward (e.g., “Would you prefer $14 today or $25 in 19 days?”). MCQ scoring involves calculating where the respondent’s answers fall in reference to discounting curves, where placement among steeper curves indicates higher levels of impulsive decision-making.

#### Emotion regulation

Emotion regulation will be assessed via Emotion Regulation Questionnaire (ERQ) [69]. The ERQ contains 10 self-report items yielding two subscale scores of emotion regulation strategies: emotion suppression and cognitive reappraisal. The ERQ demonstrates acceptable internal consistency and associations with mental health outcomes in military samples [70].

### Treatment process variables

#### Therapeutic alliance

Therapeutic alliance will be assessed with the Working Alliance Inventory-Short Form (WAI-SF) [71]. The WAI-SF has 12 self-report items that measure the patient’s perceptions about their therapist in several domains: agreement on the tasks of therapy, agreement on the goals of therapy, and development of an emotional bond. Higher scores on therapeutic alliance are associated with better outcomes in psychotherapy.

#### Treatment credibility

Treatment credibility will be assessed with the Credibility/Expectancy Questionnaire (CEQ) [72]. This instrument contains 6 items measuring the patient’s perceptions about the legitimacy of their treatment, as well as expectations for clinical improvement. Both credibility and expectancy are associated with clinical outcomes in psychotherapy. The CEQ possesses high internal consistency and strong construct validity in military samples [73].

### Covariates and descriptors

#### Traumatic brain injury

Traumatic brain injury will be assessed using the Traumatic Brain-Injury 4 (TBI-4) [74], which is a four-item self-report screening tool. It has demonstrated acceptable reliability, sensitivity, and validity with a military veteran sample.

#### Reasons for living

Reasons for living will be assessed via the Brief Reasons for Living Inventory (BRFLI) [75]. This self-report tool contains 12 items covering perceived reasons for living across six subscales: fear of social disapproval, moral objections, survival and coping beliefs, responsibility to family, child-related concerns, and fear of suicide. Many BRFLI subscales possess acceptable internal consistency and are associated with suicidal cognitions among active-duty service members [13].

### Data management plan

A copy of the full data management plan can be obtained from the corresponding author by email contact. Data will be de-identified using sequential coded ID numbers. We adopted the following data storage and transfer practices in line with prior collaborative research with NMCP and previous clinical trials conducted by members of the study team. Upon extraction from Qualtrics, data will be stored on the University of North Carolina at Charlotte (UNC Charlotte) secure cloud via a database in a password protected folder. Study team members have UNC Charlotte accounts which require password-protected, two-factor authentication to access shared databases. We will store participant identifiers separately from de-identified data in order to ensure participant responses cannot be linked. Only the study coordinator and study leadership team will have access to the list of participant identifiers. Should need arise to transfer data between institutional partners, we will use secure encrypted methods. For example, UNC Charlotte and NMCP have used DoD Safe, the U.S. military’s secure data sharing platform, in prior studies. Only trained study staff who have completed Collaborative Institutional Training Initiative confidentiality and privacy training and received IRB approval to participate in the proposed study will have access to study data. Each staff member will be permitted access only to study data that is explicitly needed for their role in the project or specific assigned task. Representatives with appropriate credentials from the DoD are eligible to review study records at any point.

### Statistical methods

#### Outcome assessment

Our primary hypotheses are that service members randomized to the G-BCBT condition will see non-inferior reductions in suicidal ideation at 6-month follow-ups compared to service members randomized to the DBT skills group condition (H1a). Similar (shorter-term) comparisons will also be examined at 3-month follow-ups. We also expect service members randomized to the G-BCBT condition will be no more likely to make a suicide attempt during the 6-months follow-up stage compared to service members randomized to the DBT skills group condition (H1b). Secondary hypotheses focus on self-regulatory characteristics like coping self-efficacy, behavioral inhibition, and emotion regulations skills. We expect that service members randomized to the G-BCBT condition will show non-inferior increases in self-regulatory characteristics at 3- and 6-month follow-ups compared to service members randomized to the DBT skills group condition (H2a). We will also explore self-regulatory characteristics as moderators of intervention effects on suicidal ideation (H2b).

To test these hypotheses, suicidal ideation as measured by the SSI will be used for the non-inferiority comparison between treatment conditions (H1a). We will use a non-inferiority threshold of 4.5 units on the SSI. Estimated treatment differences and 95% confidence intervals will be calculated. For hypothesis H1b, suicide attempts will be recorded as a secondary outcome and time-stamped through the SITBI-R over the study period. Kaplan-Meier survival curves will be extracted. A Log Rank Test will be performed to assess differences in survival curves between treatments. A Cox regression analysis will be performed and confound-adjusted hazard ratios and corresponding 95% confidence intervals will be estimated for the two treatments. Differences in survival rates during the 6-months follow-up period will be calculated.

Hypothesis H2a, which assesses differences in self-regulatory characteristics between treatment groups will be assessed at 3- and 6-month follow-ups. We will explore differences in self-regulatory characteristics between G-BCBT and DBT while accounting for between-group baseline differences. For hypothesis H2b, we will explore potential moderators to assess whether the intervention effect varies by baseline levels of the following self-regulatory characteristics: (1) Coping self-efficacy scale (across its three subscales); (2) Behavioral inhibition; and/or (3) Emotion regulation (across its two subscales). Moderators may further explain treatment efficacy heterogeneity. Additionally, treatment process variables (therapeutic alliance and treatment credibility) will be explored as covariates to assess possible associations with the primary and/or secondary outcomes. All secondary analyses will be Bonferroni-adjusted to account for multiple comparisons.

#### Secondary analyses

Data will be assessed for distributional assumptions. Any necessary transformations will be performed to meet normality distributional assumptions; or, where normality does not occur with transformation, a more appropriate method of analysis will be performed using a data-driven approach. Participant baseline characteristics will be reported. Although substantial differences between groups are not expected with the stratified randomization, we will compare baseline measures between G-BCBT and DBT groups to assure balance. If there are differences by any key characteristic(s), we will include the factor(s) as model covariates to address confounding. R version 4.0.3 statistical software will be used for analysis.

#### Handling missing data

To minimize attrition bias, we will analyze missing data patterns in advance and use appropriate statistical methods to account for missingness. Intention to treat (ITT) will be assumed among participants who had a baseline measurement but were lost to follow-up at some point during the study period. Differences in attrition by covariate or treatment will be explored and reported. Little’s Missing Completely at Random (MCAR) test will be performed to assess whether data are missing at random. Multiple Imputation by Chained Equations (MICE) will be used to impute any missing demographic covariates.

#### Sensitivity analyses

A complete case sensitivity analysis will be performed to assess the influence of multiple imputation on results. Any meaningful differences will be explored and factors associated with those differences will be identified. The suicidal ideation primary outcome has the potential to be largely zero-inflated. Thus, a sensitivity analysis will be conducted to assess the magnitude of the treatment effects through a zero-inflated multivariate regression. Similarly, covariates that are continuous or count in nature, such as age or number of prior suicide attempts, may not be linearly associated with the primary and secondary outcomes. Therefore, a generalized additive model approach will also be explored to account for potential non-linearity across such covariates. Differences in treatment effects with the primary analysis will be assessed across the covariate space.

Differences in suicide attempts will be calculated between the baseline measurement and the 9-month time point (e.g. the length of time over which the two cohorts overlap), and survival rates will be calculated and compared between the two treatments. This will account for potential differences in suicide attempts during the intervention and over a common period upon respective cohort entries. A group-based random effects sensitivity analysis will be explored, as differences in group-based delivery of treatment and group dynamics may induce between-group heterogeneity, which if identified, may be used for enhancement of intervention implementation.

### Monitoring

#### Data monitoring

There is no data monitoring committee for this trial. A research monitoring plan is not required for studies assigned a risk rating of minimal risk. There is not a plan to conduct interim analyses. In the case of unforeseen circumstances, the study leadership team (Cramer, Baker, Franks, Grover) in consultation with the NMCP IRB would make the determination whether to cease the trial.

#### Harm and auditing

Unintended impacts of the clinical trial interventions or study team interactions will be monitored through a serious adverse events and adverse events tracker. All adverse events and serious adverse events will be reported in compliance with documented reporting timelines to the NMCP IRB. Serious adverse events must be reported by phone or email within one business day of discovery and a reportable event form submitted within three business days of discovery. Adverse events that are not serious in nature, nor unexpected, nor related to the study will be reported at the annual continuing review. Corrective actions will be determined by the study leadership team in consultation with the NMCP IRB. The NMCP IRB conducts its own independent auditing of approved clinical trials to ensure adherence to study protocol procedures. We will comply with all IRB requests for audit.

### Ethics and Dissemination

#### Protocol amendments

Reporting to the NMCP IRB will occur on an annual basis as is required for IRB renewal. Protocol amendments will be submitted as needed. Appropriate training/retraining of all research staff will occur to ensure compliance with any protocol modifications.

#### Consent

The study coordinator will obtain in-person consent (see details in the recruitment section above). Information regarding use of de-identified data for secondary analyses is included in the informed consent form. Clear statements regarding the participant’s right to withdraw consent are also included in the informed consent form. A copy of the most recent consent form is available via email request to the corresponding author, and on file at Clinicaltrials.gov.

#### Confidentiality

Several mechanisms will be used to ensure participant confidentiality. Participants will be allowed to complete surveys in private venues such as a private room or office at NCMP. Additionally, 3- and 6-month follow-up data collection may be completed at home or in another chosen private location. Data collection is being conducted via secure Qualtrics administration with individualized, anonymous survey links. Data storage will occur on a credential login-required university secure storage cloud. More information can be found in the data management plan section of this paper.

#### Access to data

Members of the study university investigator leadership and analytic teams will have access to the final dataset. Researchers external to this study will be able to access deidentified data following the conclusion of the study according to procedures outlined in the data and resources sharing plan consistent with data sharing procedures set forth by the DHA.

#### Dissemination policy

Clinical trial results will be communicated in at least the following formats: (1) final report for the DoD; (2) conference paper at a military health conference; (3) peer-reviewed journal article; (4) op-ed; (5) military suicide prevention focused white paper; (6) infographic abstract, and (7) grand rounds presentation to NMCP stakeholders. Authorship for all dissemination activity will be determined using the CRedIT Taxonomy. Minimal guidelines include contribution to at least two CRedIT Taxonomy contributions including editing/approving the final output. Access to the full protocol, dataset, and code will be governed by formation of a Project Investigator Committee comprising study team members Cramer, Baker, Bryan, Gunn, and Franks. Persons seeking data, code, or protocol access will complete a request form submitted to the primary investigator (Cramer). The form will include at least persons requesting data, reason for access, IRB/regulatory information, and planned analyses. Forms will be reviewed and decisions regarding the approval of requests for proposed data, code, or protocol will be made by majority vote of the Project Investigator Committee.

## Discussion

Suicide remains a pressing public health problem in the military. BCBT has shown efficacy in an individual therapy format [7]. This clinical trial extends the promise of BCBT to address military suicide, this time in a group therapy format. An added value of the trial is understanding how theoretically [31, 34] and empirically [33, 38] relevant coping factors may impact intervention outcomes. Coping self-efficacy, emotion regulation, and behavioral disinhibition may be identified as future specific treatment targets to reduce suicide among military service members.

Results of this RCT may provide positive short- and long-term impacts in line with military needs, initiatives, and best practices. In the short term, NMCP benefits from provision of two group therapies for suicide. As previously noted, the Suicide Prevention and Response Independent Review Committee [14] summarized myriad problems precluding effective suicide prevention services in the military. To the extent G-BCBT is effective, it can provide a partial solution to problems such as clinician shortages and insufficient evaluation of suicide prevention initiatives. Also, consistent with the Navy’s recently launched Operational Stress Control program [76] that places emphasis on resilience in enhancing operational readiness, both G-BCBT and DBT boost resilience in service members by improving emotion regulation and other coping skills. These types of skills training may equip active-duty service members with effective tools for controlling and finding meaning in challenges, thereby improving operational readiness. Finally, the Department of VA/DoD [11] suicide clinical practice guidelines identify both CBT and crisis response planning as best practices. G-BCBT is an integration of both of these evidence-based practices. Offered in a group format, these best clinical suicide prevention practices can be delivered and further evaluated in a variety of military settings.

The study team has developed and refined referral processes, validated randomization and data collection procedures, and implemented a rigorous clinical fidelity assessment framework. We acknowledge a number of limitations within the clinical trial design. These include, but are not limited to, participant awareness of their intervention condition, possibility of attrition due to military deployments, and inherent biases in self-report data. Throughout the clinical trial, observed limitations will be documented; caveats and limitations to study findings will be acknowledged in all planned dissemination activity.

### Electronic supplementary material

Below is the link to the electronic supplementary material.

Supplementary Material 1 SPIRIT Checklist

## Data Availability

The datasets generated and/or analyzed during the current study are available from the primary investigator (Cramer) on reasonable request.

## Declarations

### Disclaimer

The views expressed in this presentation are those of the authors and do not necessarily reflect the official policy or position of the Department of Defense nor the U.S. Government. CDR Shawna Grover and CAPT Michael Franks are military service members. This work was prepared as part of their official duties. Title 17 U.S.C. 105 provides that “Copyright protection under this title is not available for any work of the United States Government.” Title 17 U.S.C. 101 defines a United States Government work as a work prepared by a military service member or employee of the United States Government as part of that person’s official duties.

## List of abbreviations

G-BCBT: Group-Brief Cognitive Behavioral Therapy
DBT: Dialectical Behavioral Therapy
US: United States
STBs: Suicidal thoughts and behaviors
BCBT: Brief Cognitive Behavioral Therapy
CAMS: Collaborative Assessment and Management of Suicidality
VA/DoD: Veterans Affairs and Department of Defense
CBT: Cognitive-Behavioral Therapy
DHA: Defense Health Agency
RCT: Randomized controlled trial
PTSD: Post-traumatic stress disorder
CSE: Coping self-efficacy
Project GRRIT: Project Group Risk Reduction Intervention Therapy
SPIRIT: Standard Protocol Items: Recommendations for Interventional Trials
NMCP: Navy Medical Center Portsmouth
SITBI-R: Self-injurious Thoughts and Behaviors Interview-Revised
DBTCCs: DBT-California Competency Scales
TAU: Treatment as usual
ID: Identifier
SSI: Scale for Suicide Ideation
PHQ-9: Patient Health Questionnaire-9
GAD-7: Generalized Anxiety Disorder-7
ISI: Insomnia Severity Index
BHS: Beck Hopelessness Scale
PC-PTSD-5: Primary Care PTSD Screen for DSM-5
SCS-R: Suicide Cognitions Scale-Revised
CSES: Coping Self-Efficacy Scale
MCQ: Monetary Choice Questionnaire
ERQ: Emotion Regulation Questionnaire
WAI-SF: Working Alliance Inventory-Short Form
CEQ: Credibility/Expectancy Questionnaire
TBI-4: Traumatic Brain-Injury 4
BRFLI: Brief Reasons for Living Inventory
UNC: Charlotte University of North Carolina at Charlotte
IRB: Institutional Review Board
MCAR: Missing Completely at Random
MICE: Multiple Imputation by Chained Equations
